# Labeled NSAID hypersensitivity and the risk of opioid prescribing; an observational study

**DOI:** 10.3389/falgy.2025.1611309

**Published:** 2025-07-29

**Authors:** Laila Carolina Abu Esba, Reham Alhoraibi, Salem Abu Al-Burak, Husam I. Ardah

**Affiliations:** ^1^ Pharmaceutical Care Department, Ministry of National Guard Health Affairs, Riyadh, Saudi Arabia; ^2^ King Abdullah International Medical Research Center (KAIMRC), Riyadh, Saudi Arabia; ^3^ College of Pharmacy, King Saud bin Abdulaziz University for Health Sciences, Riyadh, Saudi Arabia; ^4^ Pharmaceutical Care Department, King Abdulaziz Medical City, Riyadh, Saudi Arabia; ^5^ College of Medicine, Western University, London, ON, Canada

**Keywords:** cross-allergy, hypersensitivity, nonsteroidal anti-inflammatory drug (NSAID), opioid use, pseudo-allergy

## Abstract

**Background:**

NSAIDs are widely used for pain management but are second only to antibiotics in causing drug hypersensitivity reactions. Misclassification of these reactions often leads to unnecessary avoidance of the entire drug class, potentially resulting in increased opioid prescribing. This study aimed to assess the prevalence and characteristics of NSAID hypersensitivity, cross-reactivity patterns, and the association between NSAID hypersensitivity and opioid prescribing. The use of COX-2 selective inhibitors as a safe alternative was also explored.

**Methods:**

A retrospective cohort study was conducted at a tertiary care hospital, including patients with documented NSAID hypersensitivity between 2016 and 2023. Data on demographics, hypersensitivity reactions, NSAID cross-reactivity, and opioid prescriptions were collected. Patients with penicillin hypersensitivity were included for comparison. Logistic regression was used to analyze the association between NSAID hypersensitivity and opioid prescribing.

**Results:**

Among 319 patients with NSAID hypersensitivity, 30% (*n* = 96) were classified as true allergy, 12.5% (*n* = 40) as pseudo-allergy, and 57% (*n* = 183) were unclassified. Cross-reactivity between NSAIDs was observed in 13%, although 52% tolerated at least one other NSAID. Patients with NSAID hypersensitivity were 62% more likely to be prescribed opioids compared to those with penicillin hypersensitivity [adjusted OR 1.62 (95% CI: 1.40–1.88), *p* < 0.001]. Celecoxib was underutilized, prescribed to only 10% of hypersensitive patients.

**Conclusion:**

NSAID hypersensitivity is associated with increased opioid prescribing due to class-wide avoidance. Despite concerns about cross-reactivity, many patients can tolerate alternative NSAIDs. Improved classification tools and clinical decision support systems are needed to guide prescribers.

## Introduction

Non-steroidal anti-inflammatory drugs (NSAIDs) are among the most widely used medications for treating pain, inflammation, and fever of various etiologies (1). However, they are second only to antibiotics in causing drug hypersensitivity reactions (2–5).

NSAID induced immediate hypersensitivity reactions are internationally classified into several types: NSAID-induced rhinitis/asthma [also known as Aspirin-Exacerbated Respiratory Disease (AERD)], NSAID-exacerbated urticaria/angioedema, multiple NSAID-induced urticaria/angioedema, and single NSAID-induced reactions. These types differ in their underlying mechanisms. Single NSAID-induced reactions are immunoglobulin E (IgE)-mediated (true allergy or selective hypersensitivity), whereas the other types are mediated by cyclooxygenase-1 (COX-1) inhibition and are associated with cross-reactivity to multiple NSAIDs, including aspirin referred to as pseudo-allergy or cross-reactive hypersensitivity (6, 7).

AERD is the most common of the pseudo-allergies and is often easier to recognize due to its prominent respiratory symptoms, which can be confirmed through aspirin provocation testing. The other pseudo-allergic types are generally diagnosed based on a history of reactions to two or more different NSAIDs, typically involving cutaneous symptoms such as urticaria or angioedema. In contrast, patients with a history of reaction to a single NSAID may require a diagnostic aspirin challenge to determine the underlying mechanism (6, 8).

Because symptoms often overlap and no specific biomarker exist to confirm the exact mechanism, determining the risk of cross-reactivity between NSAIDs presents a clinical challenge. Moreover, avoidance of the entire NSAID class may unnecessarily limit analgesic options and increase reliance on opioid analgesic (9).

Recognizing the specific type of hypersensitivity is critical for predicting the cross-tolerance and guiding the selection of appropriate alternatives. However, physicians without specialized training in allergy or immunology may be unfamiliar with these classifications and diagnostic approaches. This frequently results in complete avoidance of the entire class of NSAIDs, including COX-2 selective drugs such as celecoxib, which are generally safe aleternatives (10–12).

The aim of this study was to describe the prevalence and characteristics of NSAID hypersensitivity reactions in our population, broadly categorize them as allergic or pseudo-allergic, and investigate patterns of cross-tolerance. Additionally, we assessed the utilization of COX-2 inhibitors and opioids in this population and explored the potential association between NSAID hypersensitivity and increased opioid prescribing.

## Methods

### Study design and setting

This retrospective cohort study was conducted at King abdulaziz Medical City, a tertirary care hospital in Riyadh, Saudi Arabia. The study period spanned from January 1, 2016, to January 31, 2023. Details of the instituion, its adverse drug reaction program, and realted activities have been previously described (5, 13–15).

### Study population

#### Inclusion criteria

Adults (≥18 years) with a documented allergic reaction to at least one NSAID and/or aspirin in the hospital's electronic healthcare system (EHS), BESTCare, which is described in previous publications (3).

To assess the type of hypersensitivity, a medical chart review was conducted for a sample of consecutive patients whose fisrt NSAID reaction occurred within the first six months of the study period (i.e., from January 1, 2016 to June 30, 2016). This allowed sufficient time for patients to be at risk of expossure to another NSAID and to opioids.

To assess the risk of opioid prescribing, patients with a documented penicillin allergy during the same period were included in a 2:1 ratio relative to patients with an NSAID hypersensitivity reaction, enabling a comparative analysis.

#### Exclusion criteria

Patients with delayed-onset hypersensitivity reactions were excluded, as the mechanistic pathway of such reactions exclude COX-1 inhibition type reactions and true IgE-mediated reactions, they are less common, and often more sever requiring NSAID class avoidance.

### Data collection

Demographics, clinical history, and details of hypersensitivity reactions were collected from medical records. The following variables were extracted: age at the time of chart review, generic names of all NSAIDs associated with a hypersensitivity reaction, documented symptoms of the reaction, presence of atopic conditions (e.g., asthma, allergic rhinitis, atopic dermatitis, or chronic urticaria), prescription for any other NSAID that was tolerated without any documented hypersensitivity reaction after the index NSAID reaction, and records of any opioid prescriptions.

### Definitions of hypersensitivity type

NSAID true allergy was defined using the following criteria:

1. Documented hypersensitivity to only one NSAID (other than aspirin) or two structurally related NSAIDs (see Table 1), in addition to one of the following:
  • Documented use of aspirin without any reported reaction
  • Documented use of a non-structurally related systemic NSAID without any reported reaction (excluding celecoxib and meloxicam)
2. The reaction should not be explained by any of the following:
  • Worsening of a respiratory condition in a patient with known asthma and/or rhinosinusitis
  • Urticaria and/or angioedema in a patient with a known history of chronic urticaria

**Table 1:** Classification of NSAIDs according to chemical structure (16).

Chemical Group	Drugs
Alkanones	Nabumetone (NF)
Anthranilic acids (fenamates)	Meclofenamic acid (NF), Mefenamic acid (NF)
Arylpropionic acids (Heteroaryl acetic acid)	Fenoprofen (NF), Flurbiprofen (NF), Ibuprofen, Ketoprofen (NF), Naproxen, Oxaprozin (NF)
Propionic acid derivatives	Dexibuprofen(NF), Dexketoprofen(NF), Fenoprofen (NF), Flurbiprofen (NF), Ibuprofen, Ketoprofen(NF), Naproxen, Oxaprozin (NF)
Enolic acids	Oxicams (Piroxicam, Tenoxicam) (NF)
Pyrazolidinediones	Oxyphenthatrazone, Phenylbutazone (NF)
Heteroaryl acetic acids	Diclofenac, ketorolac, Tolmetin (NF)
Indole and indene acetic acids	Etodolac (NF), indomethacin, Sulindac (NF)
Para-aminophenol derivatives	Acetaminophen (Paracetamol)
Pyrazol derivatives	Aminopyrine (NF), Antipyrine (NF), Dipyrone (NF)
Salicylic acid derivatives	Aspirin, choline magnesium trisalicylate (NF), Diflunisal (NF), Olsalazine, Salicylsalicylic acid (NF), Salsalate (NF), Sodium Salicylate (NF), SulfaSALAzine

NF, not on the institution's formulary.

### NSAID pseudo-allergy was defined using the following criteria

1. Meeting one of the following:
  • Hypersensitivity to more than two structurally unrelated NSAIDs (see Table 1)
  • Hypersensitivity to aspirin in addition to any other NSAID
2. The reaction should be explained by one of the following:
  • Worsening of respiratory condition in a patient with known asthma and/or rhinosinusitis
  • Urticaria and/or angioedema in a patient with a known history of chronic urticaria
  • New onset of urticaria or angioedema in previously asymptomatic patient
  • A combination of bronchospasm, rhinitis, urticaria, and/or angioedema in a previously asymptomatic patient

### Outcomes of interest

The outcomes included:

• Prevalence of NSAID hypersensitivity, using the number of NSAID users during the study period as the denominator
• Cross-allergy patterns, including the proportion of patients classifed as having true allergy or pseudo-allergy, and analysis of the most common cross-reactivity between NSAIDs. Utilization of COX-2 inhibitors was also assessed.
• For opioid prescribing, the incidence and frequency of opioid prescriptions were compared between patients with NSAID hypersensitivity and those with a penicillin allergy.

### Statistical analysis

The study participants were divided into three groups based on allergy classification types (pseudo-allergy, true allergy, and unclassified). Categorical data were presented as frequencies and percentages. Continuous variables were presented based on the normality of their distribution: as mean ± standard deviation for normally distributed data or as median and interquartile ranges for non-normally distributed data both overall and within each allergy classification group. Kolmogorov-smirnov normality test was used to test whether numerical data were normally distributed.

The three groups were compared using Chi-square test or Fisher's exact test for categorical variables, and ANOVA or Kruskal–Wallis test for continuous variables, as appropriate.

A multivariate logistic regression analysis was them conducted to determine whether NSAID allergy (vs. penicillin allergy) was an independent factor associated with opioid prescribing. In the model, the probability of prescribing opioids was the dependent variable, while allergy type (NSAID vs. penicillin), gender, and age were included as independent variables.

A significance level of *α* = 0.05 was used. Statistical analyses were performed using SAS 9.4 (SAS Institute Inc., Cary, NC, USA).

## Results

### NSAID hypersensitivity prevalence

Out of 635,897 patients who were prescribed NSAIDs, hypersensitivity was documented in 4,529 cases, indicating an incidence rate of 0.71% between January 2015 and January 2023. The most commonly implicated NSAIDs were ibuprofen (41.4%), diclofenac (38.2%), and aspirin (12.9%).

### Patient characteristics

The study cohort consisted of 319 patients with a documented NSAID hypersensitivity reaction, accounting for a total of 365 reactions. Among them, 67.1% were female, and the mean age was 48.4 ± 15.7 years. Approximatly 17.2% of patients had a history of asthma, and 8.2% had allergic rhinitis. The majority (79%) had no history of atopy. Table 2 presents the detailed baseline characteristics of the cohort, categorized by allergy classification (true allergy, pseudo-allergy, and unclassified).

**Table 2:** Patient characteristics, categorized by allergy classification (true allergy, pseduo-allergy, and unclassified).

Characteristic	True Allergy (*n* = 96)	Pseudo- Allergy (*n* = 40)	Unclassified (*n* = 183)	Total (*n* = 319)	*p*-value
Gender (Male)	28 (29.2%)	19 (47.5%)	58 (31.7%)	105 (32.9%)	0.1009
Gender (Female)	68 (70.8%)	21 (52.5%)	125 (68.3%)	214 (67.1%)	0.1009
Age (mean ± SD)	56.3 ± 14.55	49.9 ± 14.61	43.8 ± 14.89	48.4 ± 15.74	<.0001
History of atopy	17 (17.7%)	13 (32.5%)	37 (20.2%)	67 (21.0%)	0.1435
History of asthma	14 (14.6%)	12 (30.0%)	29 (15.8%)	55 (17.2%)	0.0711
Allergic rhinitis	6 (6.3%)	3 (7.5%)	17 (9.3%)	26 (8.2%)	0.6693
Celecoxib use after index reaction	16 (16.7%)	3 (7.5%)	13 (7.1%)	32 (10%)	0.0350

### Hypersensitivity classification

Out of the 319 patients, 30% (*n* = 96) were classified as having true NSAID allergies, 12.5% (*n* = 40) as having pseudo-allegies, and 57.4% (*n* = 183) were unclassifiable. The most commonly reported allergic reactions were to ibuprofen (41.4%) and diclofenac (38.5%), followed by aspirin (12.9%) and meloxicam (4.4%) (See Table 3: Culprit NSAID in the Index Hypersensitivity Reaction).

**Table 3:** Culprit NSAID in the index hypersensitivity reaction.

Culprit Drug	Pseudo-allergy = 40	True-allergy = 96	Unclassified = 183	Total = 319	*p*-value
Ibuprofen	15 (37.5%)	29 (30.2%)	88 (48.1%)	132 (41.4%)	0.0137
Diclofenac	9 (22.5%)	33 (34.4%)	81 (44.3%)	123 (38.5%)	0.0283
Aspirin	14 (35.0%)	19 (19.8%)	8 (4.4%)	41 (12.9%)	<.0001
Meloxicam	1 (2.5%)	12 (12.5%)	1 (0.5%)	14 (4.4%)	<.0001
Naproxen	1 (2.5%)	2 (2.1%)	3 (1.6%)	6 (1.9%)	0.8523
Lornoxicam			1 (0.5%)	1 (0.3%)	1.0000
Ketorolac		1 (1.0%)		1 (0.3%)	0.4263
Mephenamic acid			1 (0.5%)	1 (0.3%)	1.0000

### Symptoms of NSAID hypersensitivity

Patients reported a range of sympoms (see Table 4: List of Symptoms Documented with the Index NSAID Reaction). The most commonly documented sympotms included:

• Cutaneous Reactions: Urticaria (47.3%) and rash (45.8%) were the most frequently documented symproms. These typically occurred within 1–2 h after NSAID ingestion.
• Respiratory Symptoms: Shortness of breath, bronchospasm and nasal congestion were reported in approximatly 18% of cases, particulaly in patients with a history of asthma or allergic rhinitis.
• Other Symptoms: Eye/Lip swelling, general swelling, and angioedema were reported in about 13.5% of cases.
• Anaphylaxis: Anaphylactic reactions were documented in 4.7% of the cases.

**Table 4:** List of symptoms documented with the index NSAID reaction.

Sign & Symptoms	Pseudo-allergy = 40	True-allergy = 96	Unclassified = 183	Total = 319	*p*-value
Urticaria	11 (27.5%)	50 (52.1%)	90 (49.2%)	151 (47.3%)	0.0243
Rash	22 (55.0%)	42 (43.8%)	82 (44.8%)	146 (45.8%)	0.4496
Shortness of Breath	3 (7.5%)	7 (7.3%)	24 (13.1%)	34 (10.7%)	0.2565
Angioedema	1 (2.5%)	7 (7.3%)	18 (9.8%)	26 (8.2%)	0.2872
Unspecified	6 (15.0%)	5 (5.2%)	12 (6.6%)	23 (7.2%)	0.1153
Anaphylaxis		9 (9.4%)	6 (3.3%)	15 (4.7%)	0.0301
Cough	1 (2.5%)	4 (4.2%)	8 (4.4%)	13 (4.1%)	1.0000
Weakness	1 (2.5%)	3 (3.1%)	9 (4.9%)	13 (4.1%)	0.7153
General Edema	2 (5.0%)	3 (3.1%)	5 (2.7%)	10 (3.1%)	0.7274
Fever		2 (2.1%)	8 (4.4%)	10 (3.1%)	0.4650
Swelling	1 (2.5%)		6 (3.3%)	7 (2.2%)	0.1803
Bronchospasm		1 (1.0%)	6 (3.3%)	7 (2.2%)	0.4546
Myalgia		2 (2.1%)	4 (2.2%)	6 (1.9%)	1.0000
Eye/Lip swelling		1 (1.0%)	5 (2.7%)	6 (1.9%)	0.5967
Vomiting		1 (1.0%)	2 (1.1%)	3 (0.9%)	1.0000
Nasal congestion		1 (1.0%)	1 (0.5%)	2 (0.6%)	1.0000
Arthralgia		1 (1.0%)	1 (0.5%)	2 (0.6%)	1.0000
Sneezing			1 (0.5%)	1 (0.3%)	1.0000
Diarrhea			1 (0.5%)	1 (0.3%)	1.0000
Syncope			1 (0.5%)	1 (0.3%)	1.0000
Tremor			1 (0.5%)	1 (0.3%)	1.0000
Dyskinesia			1 (0.5%)	1 (0.3%)	1.0000
Tachycardia	1 (2.5%)			1 (0.3%)	0.1254

### Cross-reactivity and tolerance

Regarding NSAID tolerance, 52% of patients (*n* = 166) tolerated at least one other NSAID following their first docmented hypersentitivty reaction. The highest tolerance rates were observed with: aspirin (23.5%), and meloxicam (19.9%). However, celecoxib was underutilized as it was only prescribed in (10%) of the cases, and was significantly higher in the true allergy group (*p* < 0.035).

Cross-reactivity between NSAIDs was observed in 48 out of 365 (13.1%) reactions. The most frequent cross-reactions occurred between: ibuprofen and diclofenac (35.4%), ibuprofen and aspirin (27.1%), and diclofenac and aspirin (20.8%).

### Opioid prescribing

Among patients with NSAID hypersensitive, 58.3% (*n* = 186) recived an opioid, compared to 45.4% (*n* = 338) in the penicillin-hypersensitive group. This corresponds to a arelative risk of 1.44 (*p* < 0.05). Multivariable logistic regression, adjusting for age and sex, showed a significant association between NSAID hypersensitivty and opioid prescribing, with an adjusted odds ratio of 1.62 (95% CI: 1.40–1.88, *p*-value < 0.001). Tramdol was the most commonly prescribed opioid in both groups, followed by morphine and codeine.

## Discussion

NSAIDs remain an important culprit of drug hypersensitivity reactions, and several NSAIDs have historically been withdrawn from the market due to disproportionately higher rates of reported anaphylaxis (17). In our study, the prevalence of NSAID hypersensitivity reactions was consistent previous reports, ranging between 0.6% and 5.7% (17–20). However, our findings are based solely on documented reactions in patients' EHRs, which may underestimate the true population-based prevalence. As NSAIDs are available over counter, patients may self-medicate and potentially experience mild or moderate reactions without seeking medical attention. Furthermore, the poor documentation of patient allergies in clinical practice has been widely reported. Nonetheless, to our knowledge, this is the first study to report on the prevalence of NSAID hypersensitivity in Saudi Arabia.

Consistent with other types of drug hypersensitivity, we found NSAID hypersensitivity to be more common in females (4). In our cohort, cutaneous reactions were the most frequently documented, with urticatial rashes representing a more distinct feature of immidite hypersensitivity. However, the unspecified symptom term “rash” was also commonly documented, highlighting a documentation gap that limits accurate classification of reaction types. Respiratory symptoms were also frequent, reported in 22.8% of cases, while severe reactions such as anaphylaxis and angioedema were rarely documented. A notable proportion of patients had a history of atopy, particularly in the pseudo-allergic group, where almost 30% had a history of asthma, presumably representing cases of AERD. Interestingly, the rate of NSAID-induced respiratory symptoms in our study was lower than the 1.9% reported in a large European population-based study, suggesting that such reactions may have been under-identified in our cohort (21). Nevertheless, one of the largest EHR-based studies reported a similar distribution of symptoms (18).

Based on the definitions used in our methods, nearly 57% of reactions were unclassifiable. This highlights a major clinical challenge: deciding whether to rechallenge with another NSAID or to avoid the entire class. The lack of details on symptom onset and type in allergy documentation was the main barrier to classification. Additionally, the absence of aspirin provocation testing and limited information on subsequent NSAID use hindered accurate classification. As a result, our study had a higher proportion of reactions classified as true allergies (30%) compared to pseudo-allergy (12%), which contrasts with prior studies where pseudo-allergies tended to predominate (21–23).

Although the avoidance of drugs implicated in a patient's hypersensitivity reaction is a widely advocated and accepted practice (24), unnecessary avoidance can limit therapeutic options and result in unintended consequences, such as avoiding cephalosporins in penicillin-allergic patients, contributing to antimicrobial resistance (15). Similarly, studies have shown that up to 55% of patients labeled as NSAID-hypersensitive can tolerate another NSAID. Our findings align with this, as 52% of patients in our cohort tolerated at least one alternative NSAID, emphasizing the importance of carful evaluation before excluding the entire class.

Despite the favorable safety profile of COX-2 selective inhibitors like celecoxib, which have a very low incidence of hypersensitivity and are considered a safe alternative for both pseudo-allergic and even true-allergic patients due to its unique chemical structure compared to other NSAIDs, we found low utilization of celecoxib (only 10% of cases) in our study, despite the setting being a governmental fully drug funded setting and no restrictions to its prescribing. This finding is consistent with multiple other studies and suggests a need for increased awareness among prescribers (9, 10, 25, 26).

Given the key role of NSAIDs in the stepwise approach to pain management as an essential class before stepping up to opioids, avoiding the entire NSAID class unnecessarily can lead to increased opioid prescribing. This raises concerns about opioid overuse and the risk of opioid use disorder (OUD). Li et al. investigated this association using EHR data from a Boston healthcare system. In a cohort of patients with chronic back pain, they found that those with documented NSAID hypersensitivity had a significantly higher risk of developing OUD (odds ratio, 1.34; 95% CI, 1.07–1.67) (9). In a separate study, the same group reported higher opioid use among postpartum patients with a documented NSAID reactions (27).

In our study, we found that patients with NSAID hypersensitivity were 62% more likely to be prescribed opioids compared to those with penicillin hypersensitivity. Although the opioid epidemic has had a greater impact in the United States, similarities in clinical practice, globalization of behaviors, and rising trends in opioid misuse suggest that any region could face a similar crisis if proactive measures are not taken.

Despite the increased risk of opioid use, our cohort showed a high rate of cross-tolerability 52%. While only a 13% rate of NSAID cross-reactivity was seen in our cohort, it remains a clinically relevant challenge, given the widespread use of NSAIDs and the potential for severe outcomes. This underscores the need to improve understanding of NSAID reaction types, enhance allergy documentation, and establish clear decision-making criteria.

In line with previous EHS-integrated projects developed by our team to reduce allergy-related harm and support clinical decision-making, we are currently developing a Clinical Decision Support System (CDSS) within our EHS. This tool aims to prompt users to appropriately label NSAID reactions, classify them by type, and provide guidance on alternative therapy selection (28). The underutilization of celecoxib will also be promoted as an integral part of opioid stewardship practice and an important mitigation strategy.

Our study has several limitations. First, as a retrospective study, it depends on the accuracy and completeness of the HER documentation, which may have resulted in misclassification or underreporting. Some unclassified reactions may have included delayed-types hypersensitivity reactions. Second, confounding by indication may have occurred, as we did not adjust for pain type (acute vs. chronic), the underlying medical condition necessitating analgesia (e.g., surgical vs. trauma pain), or the use of other pain medications like acetaminophen or gabapentinoids. These unmeasured factors could potentially influence opioid prescribing patterns. However, given the high prevalence of pain conditions across the general population, the widespread use of NSAIDs, and the magnitude of the observed difference in opioid prescribing, the impact of residual confounding is likely limited. Third, the study was conducted at a single tertiary care hospital predominantly serving a Saudi population, which may limit the generalizability of findings to other healthcare settings or regions, especially considering the possible role of genetic predisposition in NSAID hypersensitivity (29). Differences in clinical documentation practices, allergy labeling behavior, prescribing patterns, access to COX-2 inhibitors across countries are among other factors that warrant further research.

While our study highlights important trends in NSAID hypersensitivity and opioid prescribing, several knowledge gaps remain. Prior studies have provided limited confirmatory data on the true prevalence of opioid substitution in patients labeled with NSAID hypersensitivity. Additionally, there is a lack of robust evidence on the frequency of cross-reactivity between selective and non-selective NSAIDs. Addressing these gaps is essential to guide safer prescribing and improve patient outcomes.

## Conclusion

Our study demonstrates that NSAID hypersensitivity is associated with increased opioid prescribing, highlighting the unintended consequences of class-wide NSAID avoidance in patients with a documented NSAID reaction and the challenges in reaction classification. The underutilization of COX-2 inhibitors suggests a need for greater prescriber awareness of safe alternatives. To address this, clinical tools like CDSS can aid in reaction classification and support informed therapeutic decision-making.

## Data Availability

The original contributions presented in the study are included in the article/Supplementary Material, further inquiries can be directed to the corresponding author.
